# Effects of the N-methyl-D-Aspartate receptor antagonist dextromethorphan on vibrotactile adaptation

**DOI:** 10.1186/1471-2202-9-87

**Published:** 2008-09-16

**Authors:** Stephen E Folger, Vinay Tannan, Zheng Zhang, Jameson K Holden, Mark Tommerdahl

**Affiliations:** 1 Department of Physical Therapy Education, Elon University, Elon, NC 27244, USA; 2 Department of Biomedical Engineering, University of North Carolina at Chapel Hill, Chapel Hill, North Carolina, 27599, USA

## Abstract

**Background:**

Previous reports have demonstrated that short durations of vibrotactile stimuli (less than or equal to 2 sec) effectively and consistently modify both the perceptual response in humans as well as the neurophysiological response in somatosensory cortex. The change in cortical response with adaptation has been well established by a number of studies, and other reports have extended those findings in determining that both GABA- and NMDAR-mediated neurotransmission play a significant role in the dynamic response of somatosensory cortical neurons. In this study, we evaluated the impact that dextromethorphan (DXM), an NMDAR antagonist, had on two distinct vibrotactile adaptation tasks.

**Results:**

All subjects, both those that ingested 60 mg DXM and those that ingested placebo, were evaluated for their amplitude discriminative capacity between two simultaneously delivered vibrotactile stimuli both with and without 3 conditions of pre-exposure to adapting stimulation. The results demonstrated that the perceptual metrics of subjects who ingested 60 mg DXM were significantly altered from that of controls when the amplitude discrimination task followed one of the conditions of adapting stimulation. Without the condition of pre-exposure to an adapting stimulus (or stimuli), there was little difference between the observations obtained from the subjects that ingested DXM and controls. Peak impact on subject response occurred at 60 min post-ingestion, whereas the scores of controls who ingested placebo were not impacted.

**Conclusion:**

The results – that DXM blocks vibrotactile adaptation – is consistent with the suggestion that NMDAR-mediated neurotransmission plays a significant role in the perceptual adaptive response. This finding is also consistent with neurophysiological findings that report observations of the effects of NMDAR block on the SI cortical response to repetitive vibrotactile stimulation.

## Background

Although the effects of conditioning stimuli – or adaptation – have been well documented in a number of sensory modalities (for review of somatosensory adaptation, see [1]; for a review of visual adaptation, see [2]), the mechanisms have not been completely elucidated. Moreover, though there are an exhaustive number of studies that have explored multiple time courses of changes in cortical response at both the population and single neuron level, there have been relatively few studies that have examined the impact of selective block of different types of neurotransmission in the cerebral cortex on sensory perception. For example, although N-methyl-D-aspartate receptor (NMDAR) block in the nonhuman primate results in decreased overall mean firing rate, a contraction of receptive fields, a reduction in the variability of the cortical response, and a decreased spatial extent in the cortical response evoked by tactile stimulation [3,4], little is known about the somatosensory perceptual correlates of NMDAR block. One finding of interest in the nonhuman primate studies is that NMDAR block results in a *decrease *in the change in responsiveness with repetitive stimulation [4]. In other words, whereas the majority of primary somatosensory cortical neurons show a significant decrease in overall mean firing rate evoked by repetitive vibrotactile stimulation, the application of NMDAR block results in the majority of these same neurons exhibiting an *absence *of this change in overall mean firing rate. In this study, we sought to observe perceptual correlates of these changes in cortical activity by characterizing the impact of NMDAR block on somatosensory adaptation.

Recently we reported that a subject's ability to discriminate between the amplitudes of two simultaneously delivered vibrotactile stimuli was highly consistent in healthy adults such that they yield similar difference limen (DL) values on this task [5,6]. Additionally, a subject's discriminative capacity was degraded systematically when one of the stimulus sites was pre-exposed to an adapting stimulus, in a duration-dependent manner [6]. The extremely low variability of the effects of adaptation on subject performance during this amplitude discrimination task suggested that this protocol could be an extremely reliable measure for the reduction of perceived intensity caused by vibrotactile adaptation. The above-referenced method of single-site adaptation, in parallel with a method that determines the impact of dual-site adaptation (both in a dual-site stimulation paradigm), were both used to obtain observations from two subject groups. One group was administered placebo and the other group was administered dextromethorphan (DXM or DM), a non-opioid over-the-counter selective NMDAR antagonist. All subjects completed the amplitude discrimination tasks prior to and after ingestion of either placebo or DXM. The purpose of this study was to investigate the effects of NMDAR block on the capacity of subjects to discriminate the amplitude of simultaneous dual-site flutter stimuli with and without pre-exposure to adapting stimulation, and the results suggest that NMDAR-mediated neurotransmission plays a significant role in the perceptual adaptive response.

## Results

Two separate 2AFC tracking protocols (see Methods) were used to obtain observations of the effects of DXM, an NMDAR antagonist, on the impact of adaptation on subjects' capacities for discrimination between the amplitudes of two simultaneously delivered vibrotactile stimuli. In the first protocol, one of the two stimulus sites that received the vibrotactile stimuli was pre-exposed to an adapting stimulus, and the tracking protocol targeted determination of the reduction in perceived intensity at the adapted stimulus site (for full details, see Methods and [6]). In the second protocol, both stimulus sites received adapting stimuli and subsequent test stimuli, but in this case, both test stimuli were identical and the difference between the adapting stimuli were tracked, based on subject response (for full details, see Methods). The working hypothesis of this protocol was that if the perceived intensities were systematically changed by the adapting stimuli, then the difference in the two adapting stimuli at the two sites would result in the subsequent test stimuli being perceived as different, with the test site receiving the larger adapting stimulus having the weaker perceived intensity.

### Results from the single site adaptation protocol

**Figure 1:**
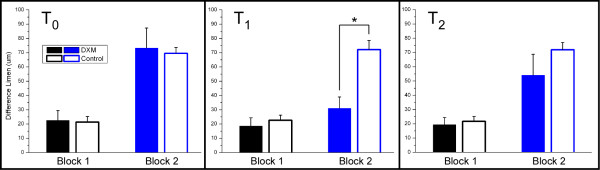
Comparison of difference limen (DL; with s.e. bars) between the Control and DXM groups for amplitude discrimination with *single-site *adaptation at times T_0, _T_1_, and T_2_. No adapting stimulus was applied to either stimulus site in Block 1, and no difference was observed between the two groups at any time for Block 1. The test condition in Block 2 was preceded by an adapting stimulus (1 sec in duration) at the site of the test stimulus. Note that at T_1_, in Block 2, performance between the groups was significantly different (* ANOVA; p < 0.01). At T_2_, Block 2 performance values for the DXM group return to baseline levels (p = 0.092).

Figure 1 summarizes the average across-subject (n = 11) performance during both blocks of the *single-site *adaptation protocol for the placebo Control (n = 9) and DXM (n = 11) groups at times T_0_, T_1_, and T_2 _(see Methods for description). Repeated measures ANOVA shows that DLs were not different between the groups at T_0 _(p = 0.54 in Block 1 and p = 0.41 in Block 2). As previously reported [6], amplitude discrimination was significantly impaired for both groups with pre-exposure to adapting stimulation (p < 0.01). One interpretation of this graph is that in Block 2, a 1 sec adapting stimulus reduces the perceived intensity of the subsequent test stimulus to the extent that a stimulus with amplitude of approximately 170 μm is now needed to be perceived nearly the same in intensity as the standard stimulus (100 μm). Note that in Block 1, without adaptation, a subject can accurately determine the difference between a 100 μm and a 120 μm stimulus that are delivered simultaneously to the two skin sites. Note that the average post-adaptation DL increased to approximately 225% above the average DL obtained in the non-adaptation block.

At time T_1 _(60 min post-ingestion) DXM drug administration had a slight effect on amplitude discrimination – though not a significant one – as indicated by Block 1 data (p = 0.57). However, in the adaptation phase of the run (Block 2) the data from the DXM group demonstrated a significantly reduced impact of adaptation compared to the control group as shown in Block 2 (p < 0.01). The average post-adaptation DL for the control group remained at a level that was over 200% above the values obtained in block 1, yet the average post-adaptation DL for the DXM group was only approximately 67% above the block 1 values. Thus, as a group, subjects that ingested DXM experienced less adaptation, and subsequently performed better at the post-adaptation amplitude discrimination task than did the control group.

Two hours post ingestion (T_2_), there was very little difference between the amplitude discrimination performance of the DXM and control group (as shown in Block 1 results). In the post-adaptation amplitude discrimination task (as shown in Block 2 results), the DXM group appeared to still be slightly outperforming the control subjects, although there were no significant differences observed between the two groups for this data set (p = 0.092), as it appears that the drug was nearing the end of its effectiveness.

### Results from the dual site adaptation protocol

**Figure 2:**
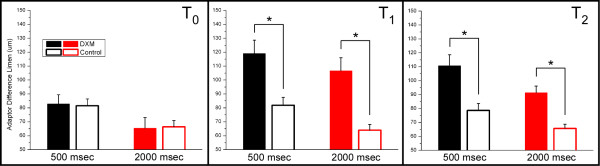
Comparison of difference limen (DL; with s.e. bars) between the control group and the DXM group for amplitude discrimination with *dual-site *adaptation at times T_0, _T_1_, and T_2 _(see Methods for description). DLs are shown for two conditions of adapting stimulus duration: 500 and 2000 msec. Note that at T_1_, performance was significantly degraded for the DXM group under both conditions of adapting stimulus duration (p < 0.01). DLs show signs of recovering to baseline levels at T_2_, although they remain significantly elevated at that time (p < 0.01).

Subject performance on the *dual-site *adaptation protocol is summarized in Figure 2. Interpretation of these results is slightly different than in the above-described data set. In this case, the "standard" adapting stimulus amplitude is constant at 50 μm and a "test" adapting stimulus initially at 150 μm is increased or decreased, based on subject response to which test stimulus felt more intense. The DL was determined by the tracked difference between the "standard" adapting stimulus and "test" adapting stimulus at which the test stimuli felt identical. With the adaptation duration of 500 msec, subjects in both groups perceived a difference between the test stimuli at an adaptor DL of approximately 80 μm. When the adaptation duration was increased to 2000 msec, the DL was reduced to approximately 65 μm. Thus, at T_0_, both groups showed significant improvement at amplitude discrimination (of identical stimuli) when the adapting stimulus duration was increased from 500 msec to 2000 msec (p < 0.01), and no difference in performance was observed between the groups for each of these two conditions (p = 0.49 and p = 0.32, respectively).

The performance observed in the drug absent condition was significantly degraded 60 min after DXM ingestion (T_1_) under both durations of adapting stimulation at (p < 0.01; Figure 2, middle panel). With DXM, subjects perceived a difference between the test stimuli at a difference in adapting amplitudes of approximately 120 μm with the 500 msec adapting stimulus duration (compare with 80 at T_0 _– approximately a 45% increase in the DL with DXM) and approximately 105 μm with the 2000 msec adapting stimulus duration (compare 65 μm at T_0 _– approximately a 66% increase in the DL with DXM). At T_2_, or 120 minutes post-ingestion, these differences between the DXM and Control groups appear to be decreasing, although they remain significantly different (p < 0.01). The contrast between these measures, obtained with the dual site adaptation protocol and those obtained with the single site adaptation protocol, suggests that the results obtained with dual site adaptation may be more sensitive in detecting differences in the impact of the DXM on adaptation.

## Discussion

In the present study, we investigated the effects of dextromethorphan (DXM), an NMDAR antagonist, on the ability of subjects to discriminate between the amplitude of two simultaneously delivered vibrotactile stimuli with/without pre-exposure to adaptation stimulation. The results show that the DXM drug administration had no effect on the subjects' performance on a standard amplitude discrimination task, but it significantly reduced the impact of adaptation on amplitude discrimination, and this reduction began to recover two hours after the ingestion of DXM.

Under control conditions (placebo instead of DXM), the observed impairment in amplitude discrimination induced by adaptation is consistent with a previously published report, which demonstrated that a subject's ability to accurately discriminate differences in the amplitude of two simultaneously delivered vibrotactile stimuli was significantly degraded with prior exposure to an adapting stimulus at the test stimulus site [6]. Tommerdahl and colleagues proposed that the effect of adaptation on amplitude discrimination capacity was due to an elevation of the detection threshold, and thus a reduction in the perceived intensity of a subsequent stimulus, induced by the adapting stimulus. This phenomenon has been characterized in a large body of psychophysical studies [7-9] that reported that when the adapting stimulus is increased in duration or amplitude, the perceived intensity evoked by subsequent test stimuli is reduced. A number of neurophysiological studies have demonstrated that the effects of reduced intensity due to adapting stimulation are possibly attributable to a reduction in the responsivity of central neurons or in synaptic processes associated with the central neurons after prolonged or repetitive stimulation. More specifically, O'Mara and colleagues [10] found that extended exposure to a vibratory stimulus produced substantial reductions in the responsivity of neurons in the cuneate nucleus, but not in the peripheral afferents. Lee and Whitsel [11] reported that repetitive brushing stimuli frequently lead individual SI neurons and neuron groups to modify their response to the repetitive afferent drive. Additionally, Lee and Whitsel [12] found that the majority (~58%) of the SI neurons sampled showed a decreased response to repetitive stimulation (3–5 Hz) of their receptive fields. In that report, it was proposed that the glutamate-mediated excitatory effects on NMDAR are to a large extent responsible for the appreciable capacities of cortical neurons to modify their physiological properties with repetitive sensory experience.

It is important to note that the spatial and temporal patterns of responses in primary somatosensory cortex (SI) to repetitive vibrotactile stimulation are largely dependent on the complex interplay of GABA-mediated inhibition [13] and NMDAR activation [14] in addition to other neural processes [15,16]. The specific contribution of NMDARs to the response evoked by non-noxious tactile stimulation has been described by relatively few studies. Duncan et al systemically administered ketamine, a noncompetitive NMDAR antagonist, in nonhuman primates and found that the responsivity, receptive field (RF) size, and mean firing rate (MFR) of SI neurons evoked by tactile stimulation were reduced in a dose- and time-dependent fashion [3]. Similarly, Whitsel et al [4] examined cortical (SI) neuron activity evoked by skin brushing stimulation and, using intravenous ketamine or phencyclidine (PCP) for NMDAR block, found the SI response to be NMDAR dependent and that SI neuron mean firing rate did not diminish with repetitive stimulation in the presence of NMDAR block. Based on their results, they proposed that in the presence of NMDAR block, normal pericolumnar interactions within the cerebral cortical networks become disrupted and the predominant excitatory drive of each SI neuron is conveyed by direct thalamocortical connections acting via AMPA receptors. However, at the same time, the response to that direct drive becomes partially suppressed by the lateral inhibitions derived from the co-activated columns that surround it, which are mediated via NMDAR on the local GABAergic interneurons. As a result, the systemic administration of an NMDAR antagonist is expected to reduce lateral inhibition on the pyramidal neurons of the directly activated column, but allow a full expression of the direct thalamocortical excitatory drive during skin stimulation. Observations obtained from 2DG, optical imaging and RF studies in the presence of NMDAR block were also consistent with these findings [13,16-19]. To summarize, the time dependent changes often observed in SI cortical response to repetitive stimulation were not observed with NMDAR block. Longer duration time dependent effects on tactile perception have also been demonstrated to be significantly impacted by NMDAR block [20].

Similar observations in reduction of adaptation to vibrotactile stimulation have been observed in autism [21,22]. However, in those studies, it was concluded that this reduction in adaptation was due to compromised or below-normal GABA-mediated neurotransmission in subjects with autism, a finding consistent with a number of reports in the autism literature ([22-28]; also see, for discussion: [23,29]). Both the reduction of the impact of adaptation on amplitude discrimination in the presence of DXM and a similar reduction observed in subjects with compromised GABA levels supports the model of dynamic cortical response to repetitive stimulation put forth by Whitsel and colleagues [15] and later expanded by Kelly and Folger [30]. One of the key elements of that model is that cortical responses in somatosensory cortex to repetitive stimulation are shaped by dynamic pericolumnar lateral interactions mediated by both GABA and NMDAR neurotransmission.

In short, without NMDAR mediated activity, the level of excitation required to evoke inhibitory responses becomes diminished, and the GABA-mediated activity required to diminish the cortical responses evoked by repetitive stimuli becomes weaker. Thus, with NMDAR block, although the overall cortical response is weaker, the change of the response caused by conditioning or adapting stimuli is significantly reduced. It should be noted that although DXM does have effects other than that of an NMDAR antagonist, the known effects of DXM as an NMDAR antagonist are consistent with the impact of NMDAR block on centrally mediated neurotransmission. The observation of weakened adaptation response – to repetitive stimulation in populations whose GABA- or NMDAR-mediated neurotransmission has been compromised – appears to strengthen the original proposal by Whitsel and colleagues that the adaptive changes observed in sensory perception at short stimulus durations (less than 5 seconds) are principally centrally mediated.

## Conclusion

The results – that DXM blocks vibrotactile adaptation – is consistent with the suggestion that NMDAR-mediated neurotransmission plays a significant role in the perceptual adaptive response. This finding is also consistent with neurophysiological findings that report observations of the effects of NMDAR block on the SI cortical response to repetitive vibrotactile stimulation.

## Methods

Twenty subjects (21–27 years in age) were studied who were naïve both to the experimental design and issue under investigation. The subjects consisted of 6 males and 5 females that received DXM during the testing session, and 5 males and 4 females that received placebo, all right-hand dominant. The study was performed in accordance with the Declaration of Helsinki, all subjects gave their written informed consent, and procedures were reviewed and approved in advance by an institutional review board.

Two separate two-alternative forced choice (2AFC) tracking protocols were used to evaluate the effects of adaptation on amplitude discriminative capacity of each subject. Each subject fasted for 2 hours prior to the study beginning. Upon arrival, the subject was seated comfortably in a chair with the left arm resting on a table surface. The subject's left hand was placed under a dual-site portable vibrotactile stimulator (CM-1; for full description, see [5]). Two probe tips (5 mm diameter) were positioned 30 mm apart along a transversally oriented linear axis along the hand dorsum. Previous studies have demonstrated that, for 25 Hz flutter stimuli, (i) the distance at which the two stimuli were positioned apart on the hand dorsum (30 mm) is well outside a subject's two point limen [31,32] and (ii) at a 30 mm probe separation there is no difference in the ability of a subject to detect a difference in the amplitudes of flutter stimulation applied simultaneously or sequentially to the 2 skin sites [5]. The hand dorsum was selected to receive the stimulation because: 1) innervation density across this skin region remains relatively constant, 2) the surface is easily accessible and permits convenient stimulator placement, 3) the surface is relatively flat, reducing confounds of skin curvature present at other potential sites of stimulation, 4) it permits positioning of the subject's arm and hand in a comfortable and stable position for the full duration of an experimental session and, 5) perhaps most importantly, there is very little, if any between-subject use-dependent changes in sensitivity at this particular site. Previous studies have demonstrated that human subjects demonstrate very consistent performance (yield similar DLs) with similar amplitude discrimination tasks on the hand dorsum [5,6,21,22,31-33]. One significant aspect of those previous studies was that consistent results were obtained although stimulus positions were randomly located on a trial-by-trial basis, and thus, the relatively large size of the probe tip apparently compensates for the differential distribution of bone vs. muscle across the hand dorsum.

Visual cueing was provided with a computer monitor during the experimental runs. Specifically, an on-screen light panel was used to indicate to the subject when the stimulus was on and when the subject was to respond. The subject was not given performance feedback or knowledge of the results during the data acquisition until all runs were completed. At the start of each run, the two probe tips were driven towards the skin until each tip registered a force of 0.1 g, as determined by a closed-loop algorithm in the CM-1 stimulator feedback system. The tips were then further indented into the skin by 500 μm to insure good contact with the skin. An audiometer was used to insure that no auditory cues were emitted from the stimulator during delivery of the range of stimuli used in this study. Practice trials were used to familiarize the subject with simultaneous two-site amplitude discrimination and correct responses on 5 consecutive trials were required before commencing with the first session. Each subject completed three experimental sessions. The first session **(T0) **was conducted prior to ingestion of any drug medication or placebo. After the first session was complete, a subject ingested either 60 mg of NMDAR antagonist DXM or a placebo. The placebo drug was perceptually similar to DXM in shape, color, texture, and taste and the subjects ingested their respective capsules with eyes closed. The study was conducted in a double-blind fashion such that neither the subject nor the experimenter was aware of the actual drug (DXM or placebo) being administered. One hour post-ingestion **(T1)**, each subject was re-tested using the same protocol. Finally, after one additional hour **(T2)**, the subjects were tested a third time to observe the effects, if any, of recovery. Each individual subject session lasted approximately 15 min (total time of 2.5 hours including experimental runtime and breaks) and the subject performed desk work, unrelated to the study, between each session. Each session consisted of two separate 2AFC protocols – one tested the effects of single-site adaptation on dual-site simultaneous amplitude discrimination (previously reported in [5,6]), and the other protocol obtained observations of the impact of simultaneous dual-site adaptation effects on an amplitude discrimination task.

### Single-site adaptation

**Figure 3:**
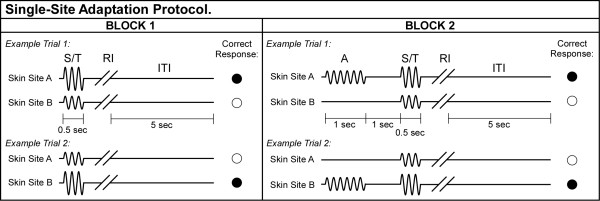
Schematic of the protocol used for amplitude discrimination with *single-site *adaptation. Two blocks of stimulus delivery were employed. In Block 1, two 25 Hz vibrotactile stimuli, the standard (S) and test (T), were delivered at the same time for 0.5 sec. A 5 sec delay (excluding subject response interval (RI)) was imposed before onset of the next trial. In Block 2, single-site adapting stimulation was first delivered for 1 sec, followed by a 1 sec inter-stimulus interval, and then the standard and test stimuli.

During the first run of each session, a tracking protocol, 40 trials total, was implemented that consisted of 2 sequential blocks (see Figure 3; not drawn to scale). This protocol has been described in detail in previous reports [5,6]. **In the first block**, a vibrotactile test stimulus (25 Hz, amplitude between 105–200 μm) was delivered to one skin site at the same time that a standard stimulus (25 Hz, amplitude fixed at 100 μm) was applied to the other skin site. The loci of the test and standard stimuli were randomly selected on a trial-by-trial basis. Stimulus duration was 0.5 sec, followed by subject response (the subject was queried to select, using a two-button switchbox, the skin site that received the most intense stimulus) and a 5 sec delay before onset of the next trial. Subjects were not provided any feedback during an experimental run. At the beginning of the experimental run, the test stimulus was 200 μm (peak-to-peak amplitude) and the standard was 100 μm. In the intial 10 trials, the difference in the amplitudes of the test and standard stimuli was adjusted on the basis of the subject's response in the preceding trial using a 1-up/1-down algorithm (the difference in amplitude was decreased if the subject's response in the preceding trial was correct; it was increased if the response was incorrect). After the initial 10 trials were completed, test stimulus amplitude was modified using a 2-up/1-down algorithm – in these trials two correct/one incorrect subject response(s) resulted in a decrement/increment, respectively, in the amplitude difference between the test and standard stimuli. This approach was selected because it enabled rapid determination ("tracking") of each subject's minimally detectable difference in the amplitudes of two-site skin flutter stimulation [5]. On average, approximately 7 reversals occurred before the conclusion of the tracking series. The step size was held constant throughout all experimental runs at 10 μm.

**In the second block (adaptation block)**, delivery of the test and standard stimuli was preceded by adapting stimulation at one of the stimulus sites. Specifically, a 25 Hz 100 μm adapting stimulus at the location of the test stimulus was delivered for 1 sec prior to the presentation of the test and standard stimuli. By presenting the adapting stimulus to the same site as the test stimulus, it was possible to quantify the effect of reduced perceived intensity due to adapting stimulation. The duration of adapting stimulation delivered was 1 sec. A 2-up/1-down algorithm was used in Block #2 to track the subject's ability to determine the most intense stimulus, and the initial conditions of Block #2 were the final conditions of Block #1.

### Dual-site adaptation

**Figure 4:**
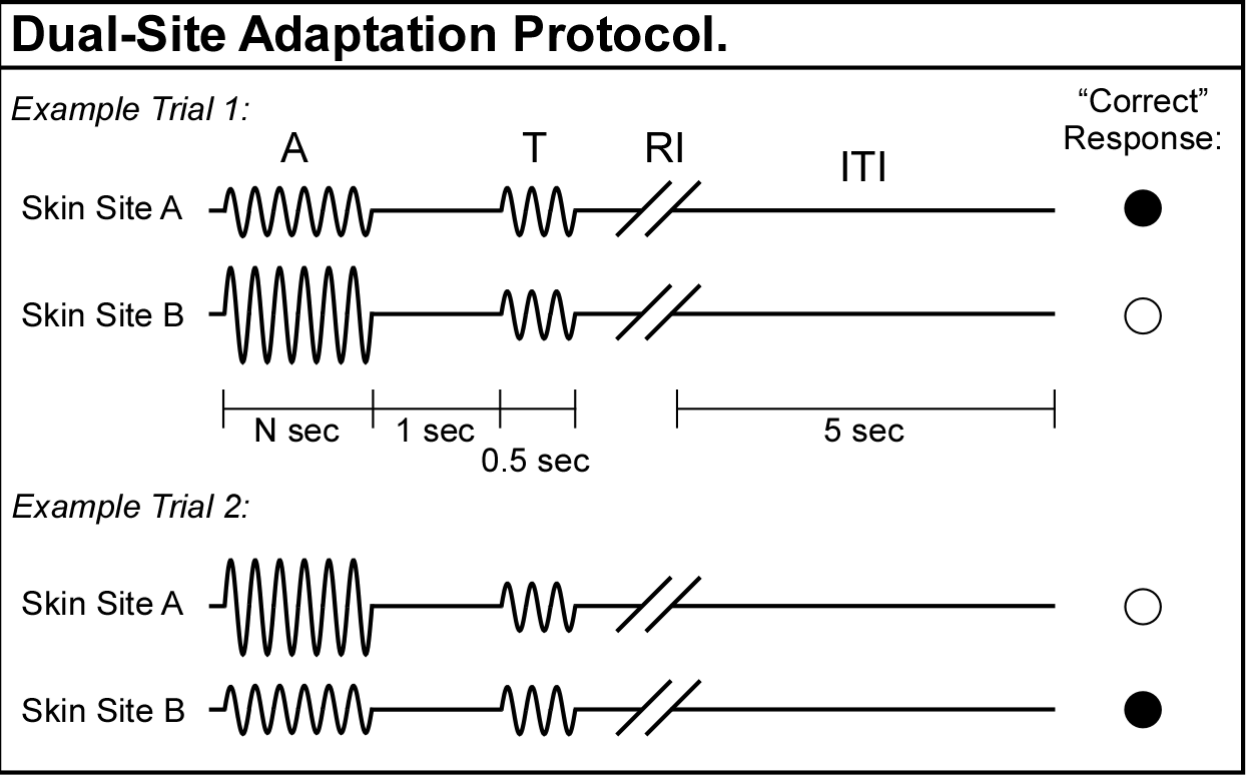
Schematic of the protocol used for amplitude discrimination with *dual-site *adaptation. During the adapting interval (A), two adapting stimuli, always of different amplitude, were delivered simultaneously to the skin, followed by a 1 sec ISI. During the test interval (T), a pair of identical test stimuli was delivered simultaneously to the same two skin sites. The subject was queried to select in each trial the skin site that received the most intense *test *stimulus. A correct response was registered when the subject chose the test stimulus that was delivered to the same location as the adapting stimulus of lower amplitude.

A separate 2AFC tracking protocol was also administered during each session (see Figure 4; not to scale). In this case, two adapting stimuli – of different amplitudes – were presented at the two stimulus sites prior to two subsequent test stimuli of equal amplitude. A schematic of example trials during the dual-site adaptation protocol is shown in Figure 4. During the adaptation interval (A), two adapting stimuli, always of different amplitude, were delivered simultaneously to the skin. One adapting stimulus was held at constant 50 μm amplitude, and the other adapting stimulus had initial amplitude of 150 μm that was varied during the experimental run dependent upon subject response. The loci of the two adapting stimuli were randomly selected on a trial-by-trial basis. The adapting stimuli were followed by a 1 sec inter-stimulus interval (ISI). During the test interval (T), a pair of identical 100 μm test stimuli was delivered simultaneously for 500 msec to the same two skin sites. During the response interval (RI), the subject was queried to select, using a two-button switchbox, the skin site that was perceived to receive the most intense stimulus. The subject response was followed by a 5 sec inter-trial interval (ITI), after which the subsequent trial began. In the first trial of the 20-trial experimental run, the variable adapting stimulus amplitude was set to 150 μm, and throughout the run this amplitude was always ≥ 55 μm. Note that a correct response was registered when the subject chose the test stimulus that was presented to the skin site to which the adapting stimulus of lower amplitude was presented. The step size was held constant throughout the dual-site adaptation experimental runs at 5 μm. Two conditions of adapting stimulus duration were implemented in separate conditional runs, randomized in order: 500 msec and 2000 msec. The working hypothesis of this protocol was that subjects would perceive a stimulus as more intense if it were delivered at the site that received the weaker of the two adapting stimuli.

## Abbreviations

DXM: dextromethorphan; GABA: Gamma-aminobutyric acid; NMDAR: N-methyl-D-aspartate receptor; DL: difference limen; 2AFC: two alternative forced-choice; ANOVA: analysis of variance; um: micron; S1: primary somatosensory cortex; RF: receptive field; MFR: mean firing rate; PCP: phencyclidine; AMPA: alpha-amino-3-hydroxy-5-methyl-4-isoxazolepropionic acid; 2DG: C14-2-deoxyglucose; ISI: inter-stimulus interval; T: test interval; RI: response interval; ITI: inter-trial interval.

## Authors' contributions

All authors (SEF, VT, ZZ, JKH and MT) contributed to the design and conduct of the experiment. VT and SEF contributed to the data analysis and the initial draft of the manuscript. All authors contributed to manuscript preparation.
